# Catalases Promote Resistance of Oxidative Stress in *Vibrio cholerae*

**DOI:** 10.1371/journal.pone.0053383

**Published:** 2012-12-31

**Authors:** Hui Wang, Shusu Chen, Juan Zhang, Francesca P. Rothenbacher, Tiantian Jiang, Biao Kan, Zengtao Zhong, Jun Zhu

**Affiliations:** 1 Department of Microbiology, Nanjing Agricultural University, Nanjing, Jiangsu, China; 2 Department of Microbiology, Perelman School of Medicine, University of Pennsylvania, Philadelphia, Pennsylvania, United States of America; 3 State Key Laboratory for Infectious Disease Prevention and Control, National Institute for Communicable Disease Control and Prevention, Beijing, China; University Medical Center Utrecht, Netherlands

## Abstract

Oxidative stress is a major challenge faced by bacteria. Many bacteria control oxidative stress resistance pathways through the transcriptional regulator OxyR. The human pathogen *Vibrio cholerae* is a Gram-negative bacterium that is the causative agent of cholera. *V. cholerae* lives in both aquatic environments and human small intestines, two environments in which it encounters reactive oxygen species (ROS). To study how *V. cholerae* responds to oxidative stress, we constructed an in-frame *oxyR* deletion mutant. We found that this mutant was not only sensitive to H_2_O_2_, but also displayed a growth defect when diluted in rich medium. Further study showed that two catalases, KatG and KatB, either when expressed in living cells, present in culture supernatants, or added as purified recombinant proteins, could rescue the *oxyR* growth defect. Furthermore, although it could colonize infant mouse intestines similar to that of wildtype, the *oxyR* mutant was defective in zebrafish intestinal colonization. Alternatively, co-infection with wildtype, but not *katG-katB* deletion mutants, greatly enhanced *oxyR* mutant colonization. Our study suggests that OxyR in *V. cholerae* is critical for antioxidant defense and that the organism is capable of scavenging environmental ROS to facilitate population growth.

## Introduction

Oxidative stress, resulting from exposure to reactive oxygen species (ROS) which can damage proteins, DNA, and membranes, is a major challenge for all living organisms. Examples of ROS include superoxide anion, hydroxyl radical, and hydrogen peroxide, generated via aerobic metabolism or production of excess amounts of ROS through phagosomal NADPH oxidase or redox-cycling antibiotics [1]. Bacteria have developed antioxidant defense systems to deal with oxidative stress by synthesizing superoxide dismutase and catalase [2]–[4]. In most bacteria, these processes are controlled by the transcriptional activator OxyR, a member of the LysR family of transcriptional regulators [5]. OxyR in *Escherichia coli* has been studied extensively. Hydrogen peroxide H_2_O_2_ activates OxyR via cysteine modification or disulfide bond formation [6]–[8]. OxyR is widely conserved among both Gram-negative and Gram-positive bacteria and numerous homologs have been shown to not only regulate the oxidative stress response, but also virulence, biofilm formation, and fimbrial synthesis [9].

*V. cholerae* is the causative agent of the devastating diarrheal disease cholera. Between epidemics, *V. cholerae* lives in natural aquatic habitats [10], [11]. Human infection normally begins with oral ingestion of food or water that is contaminated with *V. cholerae*. The bacteria are able to survive the stomach acid shock and subsequently proceed to penetrate the mucus layers of the intestinal epithelium where they adhere and colonize. As it colonizes the small intestine, *V. cholerae* produces an array of virulence factors, including cholera toxin, which is responsible for the characteristic acute diarrheal symptom of the disease. A cascading system of regulatory factors activates the coordinate expression of *V. cholerae* virulence genes [12]. *V. cholerae’s* infectious cycle is perpetuated by the resulting diarrhea, which facilitates the spread of bacteria back into the environment. [13].

The ability of *V. cholerae* to deal with oxidative stress is not well understood, however, our previous work demonstrated that *V. cholerae* can utilize its own quorum sensing systems to enhance the oxidative stress response through RpoS [14]. A similar study in *V. vulnificus* demonstrated that OxyR and OxyR-regulated catalases play important roles in oxidative stress and entering the viable but nonculturable state [15]. To further investigate the role of OxyR in the ROS response of *V. cholerae*, we constructed an *oxyR* deletion mutant and found that this mutant displayed a growth defect in aerobic rich medium as well as in the intestines of zebrafish. Two OxyR-independent catalases that were released into the extracellular milieu from living cells could restore the *oxyR* growth defect. Our study suggests that OxyR in *V. cholerae* is critical for antioxidant defense and that *V. cholerae* is capable of scavenging ROS to facilitate growth of neighboring cells.

## Materials and Methods

### Strains, Plasmids, and Culture Conditions

All *V. cholerae* strains used in this study were derived from E1 Tor C6706 [16] and propagated in Luria broth (LB) media containing appropriate antibiotics at 37°C unless otherwise indicated. In-frame deletions of *oxyR* (VC2636), *katG* (VC1560), and *katB* (VC1585) were constructed by cloning the flanking regions of these genes into the suicide vector pWM91 containing a *sacB* counter-selectable marker [17]. The resulting plasmids were introduced into *V. cholerae* by conjugation and deletion mutants were selected for double homologous recombination events. Transcriptional fusion reporters were constructed by cloning promoter sequences of the genes of study (approximately 0.5 kb sequences upstream of the start codon) into pBBR-lux which contains a promoterless *luxCDABE* reporter [18]. Plasmids containing either P*_BAD_-katG-flag* or P*_BAD_-katB-flag* were constructed by cloning *katG* and *katB* coding sequences fused with the FLAG-tag into pBAD24 [19]. P*_T7_-katG-his_6_* and P*_T7_-katB-his_6_* were constructed by cloning *katG* and *katB* coding sequences into pET30a plasmid (Novagen). The resulting plasmids were then transformed into BL21/DE3 (NEB). His-tagged protein expression and purification were performed according to the manufacturer’s instructions. Primers used in this study are listed in the Table S1.

### Culture Supernatant Preparation and Growth of *oxyR* Mutants

100 µl overnight cultures of *V. cholerae* wildtype and mutant strains indicated were spread onto LB plates and grown at 37°C overnight. LB liquid medium was then added to the plates (10 ml/plate) to collect all bacterial cells. The samples were centrifuged and the supernatants were filtered through a 0.22 µm filter. To determine the effect of cell-free culture supernatants or purified catalases on *oxyR* growth, overnight cultures of *oxyR* were inoculated 1∶1000 into fresh LB without and with 1/10 (v/v) cell-free supernatants and shaken at 37°C. OD_600_ was measured or CFU was determined at the time points indicated. In the case of CFU determination, 1/10 (v/v) cell-free supernatants prepared from wildtype cultures was included in the LB agar plates to ensure the growth of the *oxyR* mutant.

### H_2_O_2_ Disc Diffusion Plate Assay

Approximately 10^8^ bacterial cells were mixed with top LB agar (0.5%) at 42°C and poured onto solid LB agar plates. After the top agar solidified, discs saturated with 6 M H_2_O_2_ were placed in the middle. The plates were incubated at 37°C for 8 hrs and the diameter of the inhibition zone was measured for each strain.

### Measuring Catalase Transcriptional Expression Using Lux Reporters

Overnight cultures of wildtype or *oxyR* mutants containing promoter-*luxCDABE* transcriptional fusion plasmids were inoculated at 1∶20 into fresh LB containing appropriate antibiotics and shaken at 37°C until mid-log phase. A higher inoculum was used to ensure that *oxyR* mutants did not have a growth defect under such conditions. When indicated, H_2_O_2_ (50 µM) was added and all cultures were incubated for 1 hr. An aliquot of cells was then withdrawn from growing cultures and luminescence was read using a single-tube luminometer (Turner Biosystem) and normalized for growth against optical density at 600 nm. Lux expression is reported as light units/OD_600_.

### Catalase Activity Assay

Overnight cultures of wildtype, *oxyR,* or *katG/katB* mutants were inoculated at 1∶20 into fresh LB containing appropriate antibiotics and shaken at 37°C until mid-log phase. When indicated, H_2_O_2_ (100 µM) was added and all cultures were incubated for 1 hr. Rinsed cells were collected and lysed using sonication. The lysates were then subjected to catalase activity assays using the Fluorometric Catalase Activity Assay Kit (Enco Scientific) by following the manufacturer’s instructions.

### Catalase Detection and Purification

*V. cholerae* containing either P*_BAD_*-*katG-flag* or P*_BAD_*-*katB-flag* were grown on LB agar containing appropriate antibiotics and 0.1% arabinose for 12 hrs. LB liquid medium was then added to the plates (10 ml/plate) to collect all bacterial cells. The samples were then centrifuged and the supernatants were filtered through a 0.22 µm filter. Proteins in supernatants were precipitated by 10% TCA. Samples were normalized by OD_600_, i.e. cell pellets (from 25 µl of OD_600_ = 3.0 cultures) and culture supernatants (concentrated from 1 ml of OD_600_ = 3.0 cultures), and proteins were fractionated by size using sodium dodecyl sulfate-polyacrylamide gel (12%) electrophoresis. Proteins were then transferred from the gel to a nitrocellulose membrane and immunoblotted using anti-FLAG (Sigma) and anti-HapR antibody [20].

### Infant Mouse Colonization Assays

The infant mouse colonization assay was performed as previously described [21] by inoculating approximately 10^5^ *V. cholerae* cells per mouse into 6-day-old suckling CD-1 mice. After 12-hr of colonization, intestinal homogenates were collected, and the ratio of the two strains was determined by plating on LB agar plates containing appropriate antibiotics and 0.5 µM purified KatG. All strains are streptomycin-resistant and in addition, a spontaneous rifamycin-resistant *oxyR* mutant was used to facilitate selection. The results reported are from three independent experiments with two infant mice for each experiment.

### Zebrafish Colonization Assays

3-month-old zebrafish (*Danio rerio*) were placed in sterilized 1% NaCl water and 50 µg/ml streptomycin without food for 24 hrs. Approximately 10^4^/ml of mid-log-growth bacterial cells resuspended in 1% NaCl water were then added and incubated for 24 hrs. Fish were then sacrificed, rinsed, and surface sterilized by 70% ethanol. Intestines were removed, homogenized, and plated on LB agar plates containing appropriate antibiotics and 0.5 µM purified KatG. All strains are streptomycin-resistant and in addition, a spontaneous rifamycin-resistant *oxyR* mutant was used to facilitate selection. The results reported are from three independent experiments with two fish for each experiment.

### Ethics Statement

This study was carried out in strict accordance with the animal protocols that were approved by the Committee on the Ethics of Animal Experiments of the Nanjing Agricultural University. All efforts were made to minimize animal suffering.

## Results

### *V. cholerae* Culture Supernatants Rescue Aerobic Growth Defect of the *oxyR* Mutant

**Figure 1:**
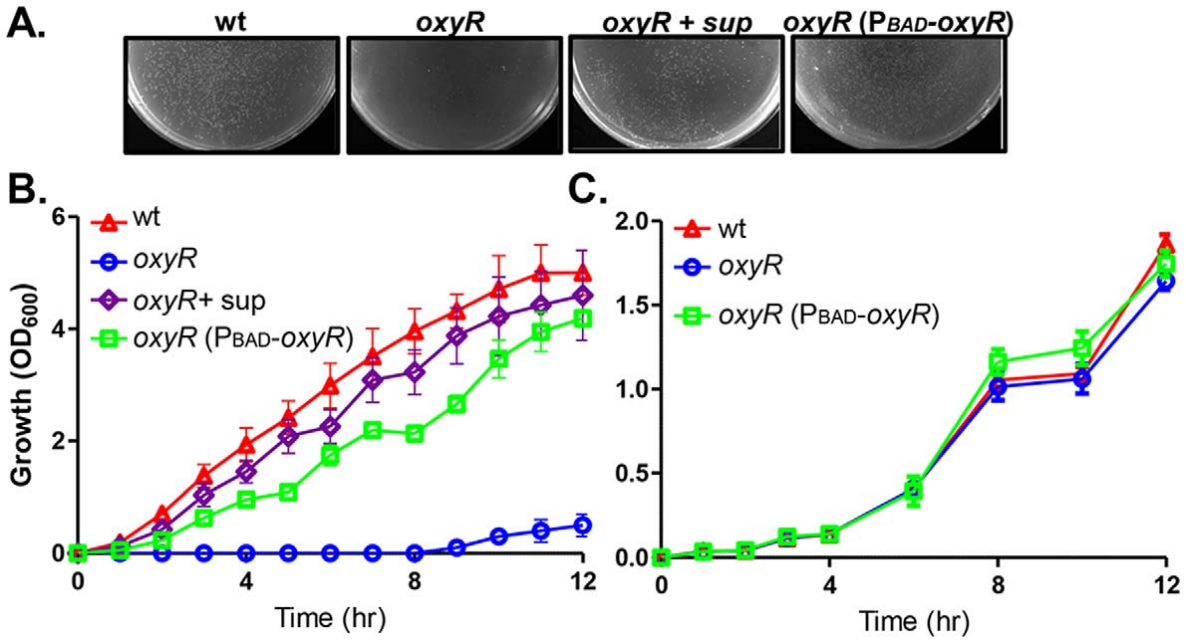
Growth of *oxyR* mutants in the absence and in the presence of stationary-phase cultural supernatants. **A.** Wildtype, *oxyR* mutants, and *oxyR* (P*_BAD_-oxyR*) grown on LB agar plates containing 0.01% arabinose without and with 1/10 (v/v) cell-free supernatants (see Methods for preparation) after overnight incubation at 37°C. **B. and C.** Wildtype, *oxyR* mutants, and *oxyR* (P*_BAD_-oxyR*) grown in LB liquid (**B**) and M9 minimal medium (**C**). Overnight cultures were inoculated 1∶1000 into fresh LB or M9-glucose medium containing 0.01% arabinose without and with 1/10 (v/v) cell-free supernatants and shaken at 37°C. OD_600_ was measured at the time points indicated. Data are mean and s.d. of three independent experiments.

Many bacteria control oxidative stress through OxyR, a LysR-type transcriptional regulator. To examine whether OxyR in *V. cholerae* is involved in the oxidative stress response, we first made an *oxyR* in-frame deletion in a *V. cholerae* El Tor strain (C6706). The *oxyR* mutant grew normally on sucrose selective medium for the double cross-over event (data not shown), but failed to form single colonies when they were re-streaked or spread on LB agar plates (Fig. 1A, center). When overnight cultures were subcultured 1∶1000 into fresh liquid LB, *oxyR* mutants could not reach high cell density even after 12-hr incubation (Fig. 1B). Expression of *oxyR in trans* restored *oxyR* mutant growth on plates and in liquid (Fig. 1A and 1B), suggesting that the growth defect of the *oxyR* mutant is in fact due to the mutation in *oxyR*. In minimal M9 medium, however, the *oxyR* mutant grew as well as that of wildtype (Fig. 1C). Furthermore, *oxyR* mutants grew poorly in LB medium but not in the minimal medium under anaerobic conditions (data not shown). It has been reported that *oxyR* homolog mutants in several Gram-negative bacteria including *E. coli*, *Xanthomonas campestris*, *Haemophilus influenzae*, and *Pseudomonas aeruginosa*, display aerobic growth defects in rich media [22]–[24]. This is not surprising since H_2_O_2_ is produced as an autoxidation product of aerobic rich broth [22] and OxyR is critically involved in oxidative stress resistance. It has been shown that addition of spent culture supernatants restore *oxyR* mutant growth in *P. aeruginosa* [22]. We therefore tested the effect of *V. cholerae* culture supernatants on *oxyR* growth. We found that in the presence of cell-free culture supernatants of wildtype *V. cholerae*, *oxyR* mutants grew normally on both solid (Fig. 1A) and liquid (Fig. 1B) LB medium. These data suggest that OxyR is critical for aerobic survival and that the *oxyR* growth defect can be rescued by addition of wildtype *V. cholerae* culture supernatants.

### Two Catalases are Involved in Rescuing *oxyR* Aerobic Growth Defect

**Figure 2:**
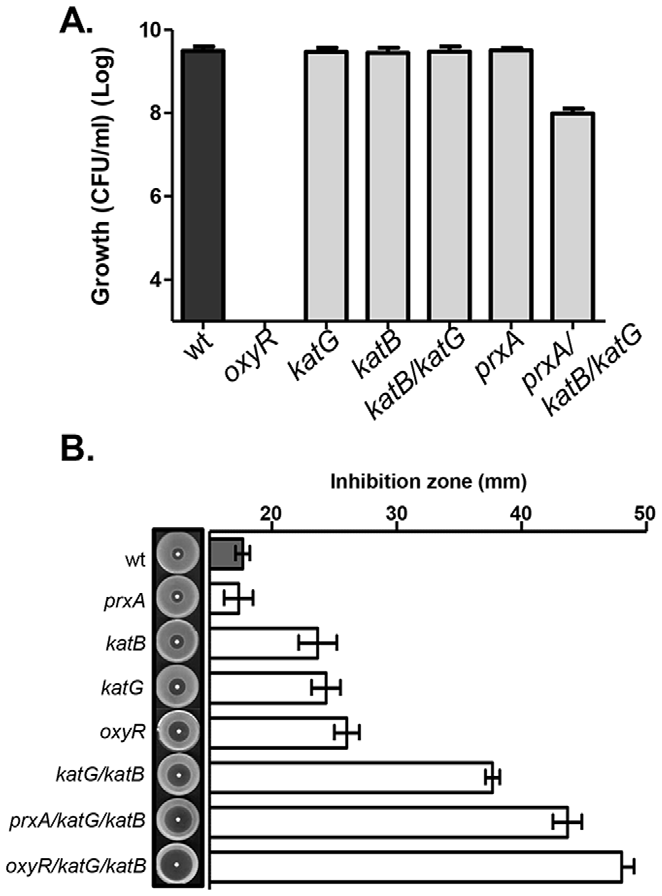
Aerobic growth and H_2_O_2_ resistance of wildtype, *oxyR*, and catalase mutants. **A.** Overnight cultures of *V. cholerae* strains were inoculated at 1∶1000 into fresh LB and shaken at 37°C for 6 hrs. Viable cells were counted by serial dilution and plating on LB agar plates containing 1/10 (v/v) cell-free supernatants prepared from wildtype cultures. **B.** Disc diffusion plate assays. Approximately 10^8^ bacterial cells were mixed with top LB agar and discs saturated with 6 M H_2_O_2_ were placed in the middle. The plates were incubated at 37°C for 8 hrs and the diameter of the inhibition zone was measured for each strain. Data are mean and s.d. of three independent experiments.

To examine possible components in *V. cholerae* culture supernatants that rescue *oxyR* growth defect, we first examined whether catalases affect *V. cholerae* growth. Two catalase genes are annotated in the *V. cholerae* genome, *katG* (VC1560), and *katB* (VC1585). Deleting either *katG* or *katB*, as well as both *katG* and *katB* together, did not affect growth (Fig. 2A). We also examined a deletion in *prxA* (VC2637), a gene that is divergently transcribed from *oxyR* and whose product has been shown to be regulated by H_2_O_2_ [25]; we found that the *prxA* mutant grew in LB similar to wildtype, whereas *prxA*, *katB*, *katG* triple deletion mutants displayed a slight growth defect.

To determine whether OxyR, PrxA, or the KatG and KatB catalases are involved in ROS resistance, we performed disc diffusion plate assays to determine the tolerance of wildtype and mutant strains to H_2_O_2_ (Fig. 2B). The mean diameter of the zone of inhibition for wildtype was 17.7 mm. The zone of inhibition for *prxA* was similar to wildtype; however, the zones around *katB*, *katG*, and *oxyR* were significantly greater than the wildtype strain. Accordingly, the double and triple deletion strains compounded the inhibitory effect caused by H_2_O_2_, and zones of inhibition of these mutants were even greater. These data indicate that these gene products must play some role in ROS resistance.

**Figure 3:**
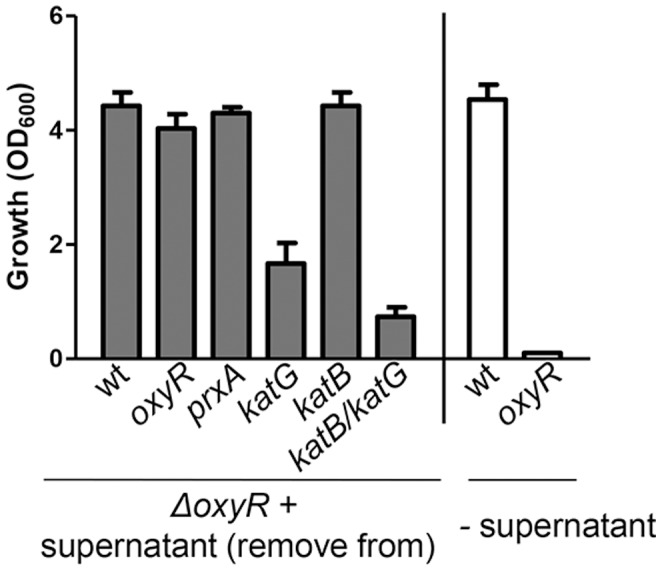
The effect of culture supernatants from different strains on *oxyR* growth. Overnight cultures of *oxyR* mutants were inoculated 1∶1000 into fresh LB with 1/10 (v/v) cell-free supernatants prepared from strains indicated and shaken at 37°C. OD_600_ was measured after 8-hr incubation. As controls, overnight cultures of wildtype and *oxyR* mutants were inoculated 1∶1000 into fresh LB without any supernatants (white bars). Data are mean and s.d. of three independent experiments.

We then prepared cell-free supernatant from cultures of different mutants and added into fresh LB to determine how *oxyR* mutants grew in “conditioned” media. Fig. 3 shows that compared to wildtype and *oxyR* mutants grown in the absence of supernatants (white bars), addition of culture supernatants of wildtype, *oxyR*, *prxA*, and *katB* could promote *oxyR* mutant growth, whereas the ability to recover *oxyR* growth in *katG* mutant supernatants was significantly reduced. The supernatant from the *katG-katB* double mutant was weaker than that of the *katG* single mutant. Taken together, these data suggest that 1) both KatG and KatB in *V. cholerae* play a role in detoxifying H_2_O_2_ and promote *oxyR* mutant growth and 2) KatG is likely more potent than KatB under the tested conditions. Interestingly, the double catalase deletion mutant supernatant could still promote *oxyR* growth to a limited extend, suggesting that other cellular components may also be involved, albeit to a lesser extent.

**Figure 4:**
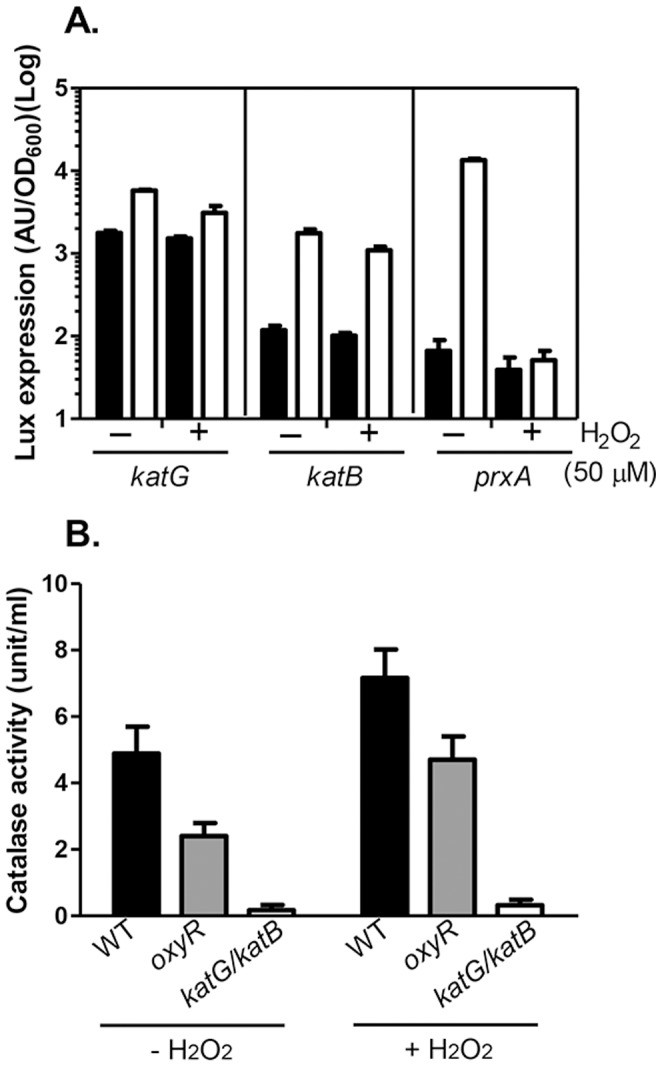
The expression and production of catalase. **A.** Overnight cultures of wildtype or *oxyR* mutants containing promoter-*luxCDABE* transcriptional fusion plasmids were inoculated at 1∶20 into fresh LB containing appropriate antibiotics and shaken at 37°C until mid-log phase. When indicated, additional H_2_O_2_ (50 µM) was added and all cultures were incubated for 1 hr. Luminescence was then measured and reported as light units/OD_600_. **B.** Overnight cultures of wildtype, *oxyR,* and *katG/katB* mutants were inoculated at 1∶20 into fresh LB containing appropriate antibiotics and shaken at 37°C until mid-log phase. When indicated, additional H_2_O_2_ (100 µM) was added and all cultures were incubated for 1 hr. The cell lysates were then subjected to a catalase activity assay. Data are mean and s.d. of three independent experiments.

To examine whether catalase genes are regulated by oxidative stress signals and OxyR, we constructed promoter-*luxCDABE* transcriptional fusion plasmids for measuring *katG* and *katB* expression. We found that both *katG* and *katB* were induced by H_2_O_2_, but interestingly OxyR was not required for the induction of these genes, at least under the growth condition tested; expression of *katG* and *katB* was only slightly lower in the *oxyR* mutant than in wildtype (Fig. 4A). As a control, we showed that *prxA*-*luxCDABE* was strongly induced by H_2_O_2_, and deletion of *oxyR* abolished *prxA* expression (Fig. 4A, right panel). These data indicate that unlike many other bacteria, neither catalase gene is regulated by OxyR in *V. cholerae*, thus explaining why the supernatant from *oxyR* mutants was able to rescue the *oxyR* growth defect.

To further confirm this, we compared catalase activity in wildtype, *oxyR*, and *katG/katB* mutants using a fluorometric catalase activity assay kit (Enco Scientific). We found that although *oxyR* mutants produced approximately 2-fold less catalase than that of wildtype (both in the presence or absence of exogenous H_2_O_2_), these mutants still produced dramatically more catalase than that of *katG/katB* mutants (Fig. 4B). These data suggest that production of KatG and KatB is independent of OxyR.

### Cell Lysis Releases Catalases to Culture Supernatants

**Figure 5:**
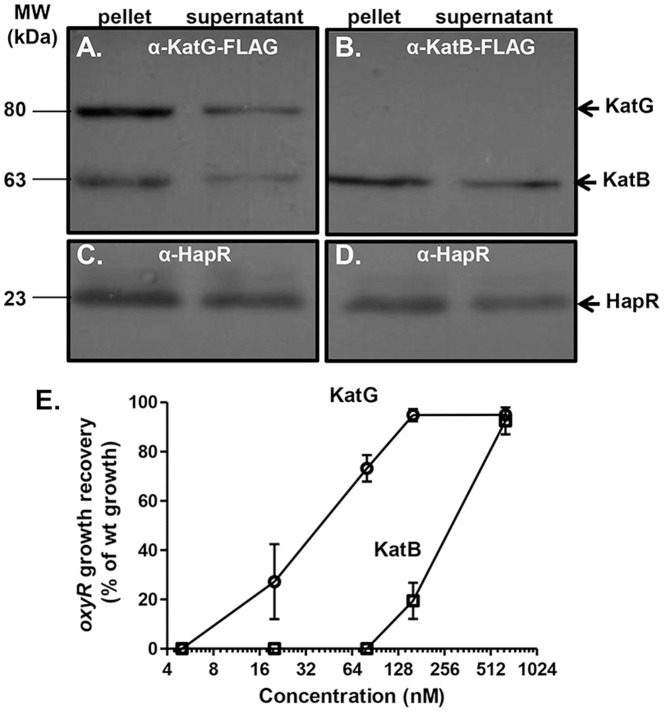
KatG and KatB localization and their *oxyR* growth promoting activity. **A–D.** *V. cholerae* containing either P*_BAD_*-*katG-flag* (**A, C**) or P*_BAD_*-*katB-flag* (**B, D**) were grown on LB agar plates containing 0.1% arabinose. Western-blot analysis to detect FLAG-tagged KatG and KatB as well as a cytoplasmic protein control HapR in the cell pellets (from 25 µl of OD_600_ = 3.0 cultures) and culture supernatants (concentrated from 1 ml of OD_600_ = 3.0 cultures). Anti-FLAG antibody and anti-HapR antibody [20] were used. **E.** Purified KatG and KatB restored the *oxyR* growth defect. Overnight cultures of *oxyR* mutants were inoculated 1∶1000 into fresh LB containing the concentrations of purified KatG-His_6_ or KatB-His_6_ indicated and shaken at 37°C. OD_600_ was measured after 8-hr incubation and compared to wildtype growth.

To determine how catalases are released into culture supernatants, we constructed recombinant FLAG-tagged KatG and KatB on a plasmid. The FLAG tag did not affect catalase functions as expression of these recombinant catalases in corresponding mutant strains restored catalase activity (data not shown). We then expressed KatG-FLAG and KatB-FLAG in *V. cholerae* and used a western blot analysis to determine the localization of catalases. We found that both KatG and KatB were presented in culture supernatants (Fig. 5A–B), however, cytoplasmic protein HapR [20], [26] was also detected in the supernatants from the same samples (Fig. 5C–D), implying that the presence of catalases in the supernatant resulted from cell lysis.

To further confirm that it is in fact KatG and KatB in the culture supernatant that rescues the *oxyR* growth defect, we purified recombinant KatG and KatB proteins. Different amounts of purified recombinant KatG and KatB proteins were added into LB medium containing diluted *oxyR* mutants and *oxyR* growth recovery was measured and compared to that of wildtype. We estimated that 128 nM KatG or 640 nM KatB could maximally rescue the *oxyR* growth to wildtype levels (Fig. 5E). These results, together with the results shown in Fig. 3, corroborates our previous finding that KatG is more efficient in detoxifying ROS than KatB.

### Physiological Importance of Catalase^+^ Cells on *oxyR* Mutants

**Figure 6:**
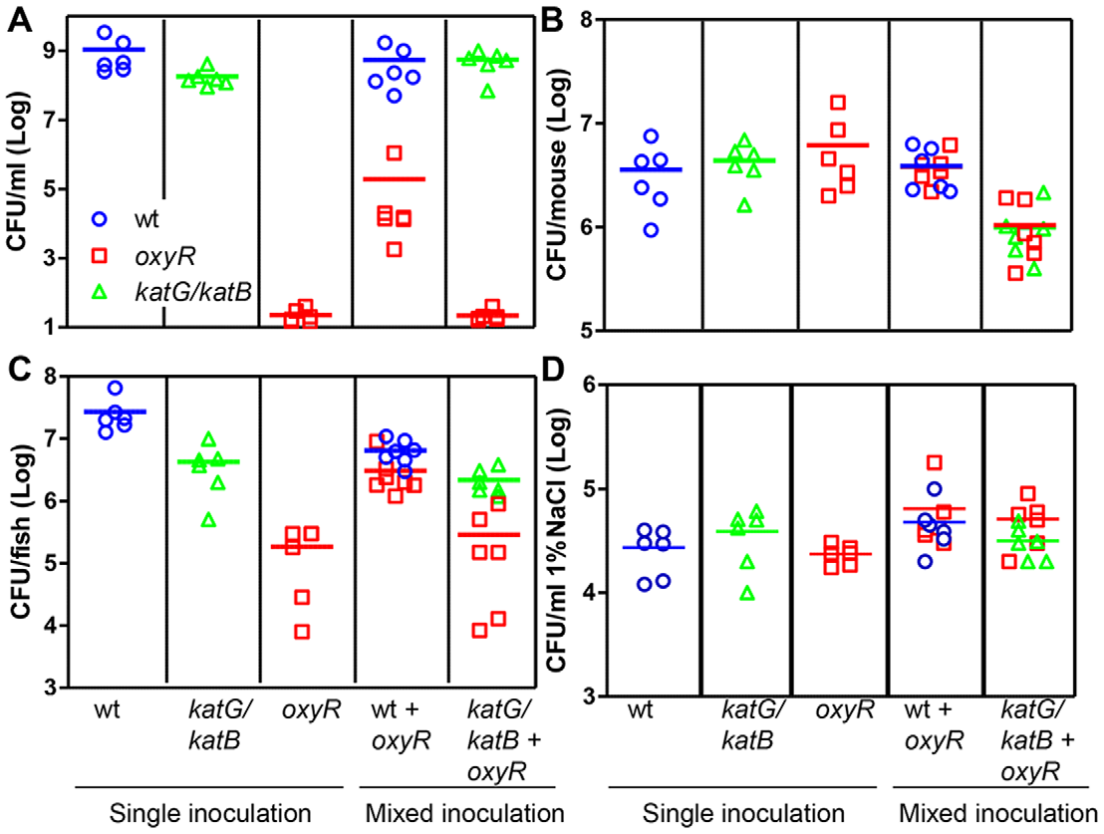
The effect of co-culturing catalase-positive and catalase-negative cells on *oxyR* growth in different environments. **A.** Overnight cultures of wildtype (circles), *katG-katB* (triangles), and *oxyR* mutants (squares) were inoculated alone or together at 1∶1000 into fresh LB medium and shaken at 37°C for 8 hrs. CFU of live cells were then determined by serial dilution and plating on LB agar plates containing appropriate antibiotics and 0.5 µM purified KatG. A spontaneous rifamycin-resistant *oxyR* mutant was used to facilitate selection. Each symbol represents CFU from one culture. **B.** Approximately 10^6^ single or mixed cells indicated were intragastrically inoculated into 6-day-old CD-1 mice. After 18-hr incubation, bacterial numbers colonized in small intestines were determined as described in **A.** The data shown are from three independent experiments and each symbol represents CFU recovered from one mouse intestine. **C and D.** 3-month old zebrafish were placed in 1% NaCl water containing approximately 10^4^/ml of bacterial cells indicated for 24 hrs. The zebrafish intestines were removed after surface sterilization by 70% ethanol and the colonization of fish intestines was determined as described in A and reported in **C.** The number of bacteria in the salt water was determined as described in A and reported in **D.** The data shown are from three independent experiments and each symbol represents CFU recovered from one fish intestine (C) or recovered from one container of salt water containing zebrafish (D).

Having shown that wildtype culture supernatants promote *oxyR* growth, we wished to determine whether metabolically active cells could also rescue growth. We first co-cultured wildtype and *oxyR* in LB medium and determined the colony forming units of wildtype and *oxyR* mutants after 6-hr growth. We found that we could recover significant numbers of *oxyR* mutants from the mixed wildtype and *oxyR* culture, whereas very few *oxyR* mutant bacteria could be recovered if they grew alone (Fig. 6A). This effect is likely due to the catalase activity of wildtype cells as the *oxyR* mutant could not be recovered from the co-culture of *katG-katB* mutants (Fig. 6A).

We then used an infant mouse colonization model [21] to examine whether OxyR is involved in *V. cholerae* pathogenesis and whether wildtype cells have any impact on the *oxyR* mutant growth *in vivo*. Fig. 6B shows that neither *oxyR* nor the *katG-katB* double mutant was defective in colonization of the infant mouse small intestine. These data indicate that at least under the conditions tested, OxyR and catalases are dispensable for *V. cholerae* colonization in mice.

In addition to the mouse model, we also examined *V. cholerae* colonization of zebrafish (*Danio rerio*). Zebrafish are amenable to host-microbe interaction studies due to their limited cost and hassle-free maintenance [27]. The zebrafish model has been used successfully for studying other Vibrio species, such as *V. anguillarum* [28], but as yet, not *V. cholerae*. We infected adult zebrafish with *V. cholerae* by static immersion of 10^4^/ml in salt water. *V. cholerae* did not cause disease in zebrafish even with a higher inoculum (data not shown), suggesting that *V. cholerae* is not a pathogen for zebrafish. Fig. 6C shows that after 24-hour incubation, on average 3×10^7^ wildtype *V. cholerae* could be recovered from each fish intestine. Single inoculation of *katG-katB* and *oxyR* mutants displayed approximately 7-fold and 16-fold reduction of colonization, respectively, as compared to wildtype (*p* value <0.01). No significant difference was observed between different strains grown outside the fish in 1% NaCl salt water (Fig. 6D). Interestingly, when the *oxyR* mutant was coinoculated with wildtype, but not *katG-katB*, colonization of *oxyR* mutants increased significantly as compared to that of single-inoculated *oxyR* (*p* value = 0.028). These data suggest that catalases and OxyR are important for *V. cholerae* colonization of zebrafish intestines and that the *oxyR* colonization defect can be overcome by co-infecting with wildtype cells. It is unclear why *oxyR* mutants displayed a defect in colonization of zebrafish intestines but not infant mouse intestines. It is possible that these two host environments may differ in the amount of ROS generated as antimicrobial agents.

## Discussion

*V. cholerae* is an opportunistic human pathogen that has two distinct life styles: in aquatic environments, often associated with plankton, zooplankton, and other marine organisms, and propagating in human small intestines [29]. In both environments, however, oxidative stress induced by reactive oxygen species (ROS) must be a common stress condition to which *V. cholerae* encounters. For example, in marine systems, the absorption of solar radiation, together with dissolved organic matter in seawater, leads to the photochemical production of diverse reactive transients, including ROS [30]. ROS are also produced as part of the host innate immune response to kill invading bacteria. The NOX oxidase family of enzymes is responsible for ROS production in the host. The recently discovered dual oxidase (DUOX) generates H_2_O_2_ at the apex of mucosal cells [31], while DUOX2 is expressed along the digestive tract [32]. In addition, the intestinal mucosa is constantly exposed to luminal oxidants from various sources [33], [34]. In order to survive, many pathogens produce enzymes capable of detoxifying ROS [35]. In this work, we aimed to identify the role of the transcriptional activator, oxyR, in *V. cholerae* ROS survival. The expression of oxidative stress response, virulence, biofim formation and fimbrial synthesis genes is often controlled by OxyR in many bacteria. The above results indicate that, like its orthologs, *V. cholerae* OxyR is also critical for oxidative stress resistance; we have demonstrated that the *oxyR* null mutation is hyper-sensitive to H_2_O_2_ exposure (Fig. 2B). Those genes under OxyR control and the molecular mechanisms of OxyR regulation under oxidative stress are currently under investigation.

*V. cholerae oxyR* mutants could not form single colonies on LB plates, and when a small inoculum was used, *oxyR* mutants failed to grow in LB liquid medium (Fig. 1) even though the expression and production of catalases KatG and KatB is not dependent on OxyR (Fig. 4). This defect could be overcome by addition of cell-free culture supernatants from wildtype as well as *oxyR* mutants, but not from catalase mutants (Fig. 3). This is not surprising, nor unique to the *oxyR* mutant of *V. cholerae* [22]–[24]. OxyR in *E. coli* has been shown to not only activate *katG*, but also *ahpCF* (encoding alkyl hydroperoxidase), *dps* (a nonspecific DNA-binding protein), *gorA* (glutathione reductase), *grxA* (gluaredoxin) and other oxidative defense related genes [36]. Although it has been reported that OxyR is required to induce the expression of the major catalase KatA in the *P. aeruginosa* strain PA14 [37], in the PAO1 strain, KatA is present in *oxyR* culture supernatants, and overexpression of OxyR-controlled AhpB and AhpCF partially rescues the aerobic growth defect but not H_2_O_2_ resistance of *oxyR* mutants [22]. Moreover, although the expression of *katG* and *katB* was not controlled by OxyR in *V. cholerae*, the *oxyR* mutant did produce less catalase than that of wildtype (Fig. 4B), suggesting that other catalase or catalase-like proteins are present in *V. cholerae*. Taken together, we speculate that the reason that *V. cholerae oxyR* mutants have an aerobic growth defect in rich medium is that OxyR may be required for expression of a number of genes that are critical for oxidative stress, even though catalases are still produced in *oxyR* mutants. Addition of excess amounts of catalases, however, can scavenge ROS produced during the growth of *oxyR* mutants.

Our data are also suggestive of cooperativity in *V. cholerae* populations. We found that mixed inoculation of *oxyR* mutants with wildtype but not *katG-katB* mutants in LB medium greatly stimulated *oxyR* growth (Fig. 6A), indicating that constitutively produced catalases, either from living or lysed cells, could help neighboring bacteria scavenge ROS, ultimately increasing the overall fitness of the bacterial population. This phenomenon was observed in the zebrafish colonization model (Fig. 6C). The *oxyR* mutant infection and survival was significantly increased when co-infected with wildtype bacteria. Although *V. cholerae* is not a natural pathogen for zebrafish, it has been reported that zebrafish gut microbiota contain Vibro species [38], including *V. cholerae* (J.F. Rawls, personal communications). The importance of OxyR in *V. cholerae* colonization of zebrafish intestines and the cross-protective properties of KatG and KatB exemplify the importance oxidative stress resistance plays in *V. cholerae* life cycles.

## Supporting Information

Table S1 **Primers used in this study.**
(PDF)
