# Supplementary material for: Catalases Promote Resistance of Oxidative Stress in Vibrio cholerae
Source: PLoS One. 2012 Dec 31;7(12):e53383. doi: 10.1371/journal.pone.0053383 (PMC3534063; doi:10.1371/journal.pone.0053383)
Supplement: Table S1 — Primers used in this study. (PDF) [file pone.0053383.s001.pdf]

Table S1. Primers used in this study.

Name	Sequence
***oxyR* in-frame deletion**	
oxyR-1	5' ATAGCGGCCGCCTTGCACCTTGTATTGAGGG 3'
oxyR-2	5' CGAGTCGACGACGGATCTGGCCACTCAAGG 3'
oxyR-3	5' CCAGATCCGTCGTCGACTCGTCTTGGCTTA 3'
oxyR-4	5' TATCTCGAGGCCGAGCAAGACGCAGCAAG 3'
***prxA* in-frame deletion**	
prxA-1	5' ATAGCGGCCGCTTTGCCCTCTTCGAGCTGAC 3'
prxA-2	5' CCGCACCAAGACCCGGCAAGCTAAACACGA 3'
prxA -3	5' CTTGCCGGGTCTTGGTGCGGAGATGATGGG 3'
prxA -4	5' CAACTCGAGGTCGTTTACGCCAAGGCAAG 3'
***katG* in-frame deletion primers**	
katG -1	5' ATAGCGGCCGCTAATACGGCGTGGCACTTCG 3'
katG -2	5' CATAAGCACGGTTTAGCGCTTTAGGCCACC 3'
katG -3	5' AGCGCTAAACCGTGCTTATGCGGAAGTGTA 3'
katG -4	5' ATACTCGAGGCCGCTGCATGATTACTTGG 3'
***katB* in-frame deletion primers**	
katB -1	5' ATTGCGGCCGCGCGGTAGATCTCTTGTTCAC 3'
katB -2	5' CCAGAACCACTCATTTGAGGCAATGCCGGC 3'
katB -3	5' ATTGCCTCAAATGAGTGGTTCTGGGCTATG 3'
katB -4	5' TAACTCGAGGTGCCGCTCGATTGAAGAAC 3'
**Promoter-*luxCDABE* reporter constructs**	
prxA - PvuI	5' GCGCGATCGCAGCTCCAATTCAGGGAAACG 3'
prxA - SpeI	5' GCGACTAGTCCTGTGGAATGGTTTGACCTTCT 3'
katG - PvuI	5' GCACGATCGGACTTAGCCATAGATGACCTCTCTC 3'
katG - SpeI	5' GCAACTAGTGTTTAGCGCTTTAGGCCACCAG 3'
katB - PvuI	5' GCGCGATCGTGCTCGCAATGCCAGAGTAATAAG 3'
katB - SpeI	5' GCGACTAGTTGCCCAGCGAGAATATAAGGGTA 3'
**His6-tag constructs**	
katG-F	5' CAGCGCGCCATATGGAGCACAATAAAGCGGGTTCAAG 3'
katG-R	5' CAGCTCGAGCACCAGATCAAATCGATCCGCATTC 3'
katB-F	5' CAGCGCCGATTAATTTGATAAGTAAAAATATGAGAAGTCATCTAAA 3'
katB-R	5' TATCTCGAGCATCGCGGCCAGTTTTGCCAC 3'
**FLAG-tag constructs**	
katG -F	5' GCGGAATTCATGGAGCACAATAAAGCGGGTTCAAGC 3'
katG-flag-R	5' GCGGTCGACTTACTTGTCGTCGTCGTCCTTGTAGTCCACCAGATCA AATCGATCCGCATTC 3'
katB -F	5' GCGGAATTCTTGATAAGTAAAAATATGAGAAGTCATCTAA 3'
katB - flag-R	5' ATCGTCGACTTACTTGTCGTCGTCGTCCTTGTAGTCCATCGCGGCCA GTTTTGC 3'
***oxyR* complementation constructs**	
oxyR-F	5' GCCGAATTCATGAACATTCGTGATTTTGAATAC 3'
oxyR-R	5' GCGTCTAGATTACTCGCTTTGCTGTAAGCG 3'
